# Complete Mitogenome sequencing of the fish louse *Argulus japonicus* (Crustacea: Branchiura): Comparative analyses and phylogenetic implications

**DOI:** 10.3389/fvets.2024.1376898

**Published:** 2024-03-25

**Authors:** Lidan Wang, Zun Hu, Zhao Wang, Pengchen Zhu, Guoshan Wei, Xinyi Fan, Jiali Huang, Ruixi Wang, Hui Wang, Yue Xie

**Affiliations:** ^1^Department of Parasitology, College of Veterinary Medicine, Sichuan Agricultural University, Chengdu, China; ^2^Department of Food Technology and Science, College of Food Science, Shanghai Ocean University, Shanghai, China

**Keywords:** *Argulus japonicus*, fish lice, genetic markers, mitogenomes, phylomitogenomics

## Abstract

The fish louse *Argulus japonicus*, a branchiuran crustacean of the Argulidae family, is attracting increasing attention because of its parasitic tendencies and significant health threats to global fish farming. The mitogenomes can yield a foundation for studying epidemiology, genetic diversity, and molecular ecology and therefore may be used to assist in the surveillance and control of *A. japonicus*. In this study, we sequenced and assembled the complete mitogenome of *A. japonicus* to shed light on its genetic and evolutionary blueprint. Our investigation indicated that the 15,045-bp circular genome of *A. japonicus* encodes 13 protein-coding genes (PCGs), 22 transfer RNAs (tRNAs), and 2 ribosomal RNAs (rRNAs) with significant AT and GC skews. Comparative genomics provided an evolutionary scenario for the genetic diversity of 13 PCGs: all were under purifying selection, with cox1 and nad6 having the lowest and highest evolutionary rates, respectively. Genome-wide phylogenetic trees established a close relationship between species of the families Argulidae (Arguloida) and Armilliferidae (Porocephalida) within Crustacea, and further, *A. japonicus* and *Argulus americanus* were determined to be more closely related to each other than to others within the family Argulidae. Single PCG-based phylogenies supported nad1 and nad6 as the best genetic markers for evolutionary and phylogenetic studies for branchiuran crustaceans due to their similar phylogenetic topologies with those of genome-based phylogenetic analyses. To sum up, these comprehensive mitogenomic data of *A. japonicus* and related species refine valuable marker resources and should contribute to molecular diagnostic methods, epidemiological investigations, and ecological studies of the fish ectoparasites in Crustacea.

## 1 Introduction

The fish louse *Argulus japonicus*, a branchiuran crustacean belonging to the family Argulidae, has garnered considerable attention due to its parasitic tendencies in various fish and significant threats to global fish farming (1, 2). This parasite is often found on the caudal fin, skin, and chin of infected fish, and its lifecycle typically involves egg, larval, and adult stages (3). After mating, female lice detach from the host and lay eggs on hard substrates in the aquatic environment. These eggs hatch into free-swimming larvae in 10 days at 35°C, and then the larvae actively seek out suitable fish hosts, initiate the parasitic phase, and develop into adults (1, 4). Morphologically, the adult has a conspicuous carapace and a pair of specialized appendages for clinging to the fish. During this attachment, the parasitic louse uses its mouthpart to penetrate the integument of fish and feed on the host blood and tissue fluids (5, 6). In fish farming, infections with *A. japonicus* can cause ulceration and immunological suppression, as well as secondary infections with bacteria and fungi (1, 5). Furthermore, this parasite can also act as a carrier to transmit fish pathogens, such as the spring viremia of the carp virus and dracunculoid/skrjabillanid nematode larvae (3). Increased epidemiological evidence shows that *A. japonicus* is becoming prevalent throughout many countries (4, 7, 8). For example, Avenant-Oldewage reported a high infection rate of *A. japonicus* in the Olifants River system of South Africa and Mozambique (9). Wafer et al. documented the frequent presence of *A. japonicus* in goldfish (*Carassius auratus*) from Florida, Georgia, Louisiana, California, Hawaii, Illinois, Maryland, Wisconsin, Washington, and Texas in the United States (10). Furthermore, the occurrence and prevalence of *A. japonicus* were recently recoded in some Asian countries, including Japan, Indonesia, Turkey, Pakistan, and Iran (7, 8, 11–13). In China, Alsarakibi et al. demonstrated a 22.3 ~ 47.8% prevalence of *A. japonicus* across Chinese rivers, fish farms, and ponds and emphasized the lack of approved drugs for its control in China (6, 14). Furthermore, numerous studies revealed a strong capability of *A. japonicus* to adapt to new surroundings, including its hosts (e.g., Amur catfish, black carp, brown trout, big-scaled redfin, and mandarin fish), suggesting its natural widespread and occurrence in various aquatic niches caused by the rapid evolution under ecological pressure rather than introduction by hosts (4, 15–18). Combined, these studies show an emerging concern for how to advance surveillance and control of *A. japonicus* in global fish farming.

Traditionally, *A. japonicus* surveillance relies on morphology-based identification and differentiation (5, 19–21). However, such taxonomic scrutiny often faces challenges due to the need for experienced microscopists to accurately identify and distinguish *A. japonicus* from other related species, especially at the larval stages (1, 3). Therefore, obtaining a more efficient and reliable way to identify and differentiate *A. japonicus* or larvae has become crucial for field diagnosis and epidemiological investigation, and achieving this goal is foreseeable only through the utilization of molecular approaches. Recently, molecular tools employing genetic markers from the nuclear and mitochondrial (mt) DNA offer a fast and sensitive approach to unveiling the genetic makeup and phylogenetic relationships of targeted species and have been widely used for species-specific identification and differentiation among various organisms, including *Argulus* (13, 19, 22–25). For example, nuclear genetic markers, including small ribosomal protein 18 (18S), OPC19, and OPH11, have been employed to explore the genetic diversity and species identification of *Argulus* parasites (6, 19, 26, 27). Moreover, the mt genes, such as cox1, nad1, and nad4, have also proven effective for *Argulus* identification because of their matrilineal inheritance, high copy number, lack of recombination, and rapid evolution (28–31). However, compared to single or partial genetic loci, a complete mt genomic dataset would be especially powerful for displaying sufficient interspecies variability and describing species specificity (32, 33). Unfortunately, a sequence search against GenBank revealed that there has been no information available on the complete mitogenome of *A. japonicus* so far. In this study, it was designed to sequence and assemble the entire mitogenome of *A. japonicus* using Illumina technology. Combined with genome annotation, comparative mitogenomics, and phylomitogenomics, our comprehensive molecular characterizations would refine the understanding of the genetic and evolutionary blueprint of *A. japonicus* and contribute to diagnostic methods, epidemiological investigations, and ecological implications of *A. japonicus* and related crustacean species.

## 2 Materials and methods

### 2.1 Sample collection, DNA extraction, PCR amplification, and sequencing

Between March and July 2023, a total of 530 cultured and wild fish samples were obtained from rivers and fish farms in Sichuan, China, using diverse methods, including angling, purse-seining, gill-netting, and trapping. Following capture and labeling, a meticulous examination for ectoparasites was conducted on the external surface of each fish using a hand lens. Approximately 63 *Argulus* specimens were harvested, preserved in 90% ethanol, and transferred to the parasitological laboratory of Sichuan Agricultural University (Chengdu, China) for morphological identification using the taxonomic keys of Wadeh et al. (34). Ten specimens were morphologically identified as *A. japonicus*, and then two specimens were chosen for further molecular identification by PCR amplifying and sequencing the 18S (19), followed by comparison with the previously documented *A. japonicus* sequence (GenBank accession number: MW857091). A result of 100% sequence identity of the 18S between both specimens and *A. japonicus* confirmed their species identity.

### 2.2 Genome sequencing, assembly, and annotation

To decode the *A. japonicus* mitogenome, the total genomic DNA (gDNA) was extracted from six louse specimens using the MiniBEST Universal Genomic DNA Extraction Kit Ver.5.0 (TaKaRa, Dalian, China). Following gDNA yield and integrity examination, a 0.2-μg aliquot of gDNA was fragmented, end-paired, and ligated to adaptors. The ligated fragments were isolated on agarose gels and amplified by PCR to produce the Illumina TruSeq library. A 300-bp paired-end (PE) library was constructed and sequenced. Approximately 3.2 Gb clean data emerged after quality-trimming short and low-quality reads with poly-Ns (>15 bp Ns) or > 75 bp bases with a quality score ≤ 3. These reads were further assembled with IDBA-UD using the following parameters: similarity threshold of 98% and minimum and maximum *K*-values of 80 and 240 bp, respectively (35). Genome assembly validation was achieved by mapping clean reads onto the acquired mitogenome sequences with Geneious v10.1.3 (36). Simultaneously, the assembled mitogenome was also confirmed by PCR, which amplified eight overlapping fragments (sizes ranging from 1.8 to 2.5 kb). These fragments were chosen based on the conserved region alignments of available *Argulus* mitogenomes, and their corresponding PCR primers are shown in Supplementary Table S1. All PCR reactions were conducted in a 25 μl volume, containing 2 μl of gDNA, 10 μl of 2 × TransTaq® HiFi PCR SuperMix (TaKaRa), 1.5 μl of each primer (10 pmol each), and 10 μl of ddH_2_O. Reaction conditions comprised 4 min denaturation at 94°C, followed by 35 cycles of 40 s at 94°C, 45 s at 2 ~ 3 min at 68°C, adhering to Tm values and product lengths, with a final extension at 68°C for 10 min. The PCR products were analyzed by agarose gel electrophoresis, and target amplicons were sequenced directly or after sub-cloning into the pMD19-T vector (TaKaRa). Each amplicon was triply sequenced for accuracy. A combination of manual alignments and online BLAST was used to annotate the final *A. japonicus* mitogenome (37, 38). The circular mitogenome map creation was achieved with MacVector v18.6.^1^ The complete *A. japonicus* mitogenome was deposited in GenBank under accession number: PP190482.

### 2.3 Sequence analyses

Using the open reading frame (ORF) finder and Primer Premier v5.0 (39), the amino acid sequences of protein-coding genes (PCGs) of the *A. japonicus* mitogenome were inferred using the invertebrate mt genetic code. Codon use profiles were examined using MEGA v11 (40). Additionally, the nucleotide skewness of the *A. japonicus* mitogenome was assessed through the following formulas: AT skew = (A − T)/(A + T) and GC skew = (G − C)/(G + C) (41). Alignments of the nucleotide and amino acid sequences of each PCG and concatenated PCGs of *A. japonicus* and other crustaceans were executed with MEGA. Based on pairwise alignments, the nucleotide and amino acid sequence identities were computed with DNASTAR v17.1.1.^2^ Synonymous (Ks) and non-synonymous (Ka) substitution rates were calculated using DnaSP v6.12.03 (42), and genetic distances were measured with MEGA using Kimura-2-parameter (K2P).

### 2.4 Phylogenetic analyses

To determine the classification positions of *A. japonicus* in the family Argulidae and of the family Argulidae within Crustacea, 29 mitogenomes of crustaceans were retrieved from GenBank (Supplementary Table S2). Phylogenetic relationships were deduced on the basis of either a concatenated amino acid dataset of 13 PCGs or an individual amino acid dataset of each PCG. During the procedures, sequence alignments were achieved using T-Coffee v7.81^3^ and the ambiguous regions were removed using GBLOCKS v0.91b.^4^ Phylogenetic analyses were carried out with maximum parsimony (MP) and Bayesian inference (BI) using *Calanus hyperboreus* as the outgroup. In brief, the MP analysis was constructed through PAUP* (43) using either concatenated or individual PCG datasets. The equally weighted parsimony, together with heuristic searches with tree-bisection-reconnection (TBR) branch-swapping and 1,000 replicates of Wagner trees, were executed. Five trees per replication were sampled, and the optimal tree was chosen using the Kishino–Hasegawa method. Bootstrap resampling was computed for each nodal support. For the BI analysis, the phylogenetic trees were reconstructed with MrBayes v3.2.7a^5^ using four independent Markov chains, running for 40,000,000 (concatenated PCG dataset) and 3,000,000 (single PCG dataset) metropolises coupled Monte Carlo generations. Trees were sampled every 40,000 and 3,000 generations. Once the average standard deviation (SD) of the split frequencies dropped below 0.01, the first 25% of trees were discarded as “burn-in,” and the remaining were used to compute Bayesian posterior probabilities (PPs). The evolutionary distance was estimated using the MrBayes order (aamodelpr = mixed) with default parameters. A consensus tree was obtained and visualized using Treeview X.^6^

## 3 Results and discussion

### 3.1 General feature of *Argulus japonicus* mitogenome

**Figure 1:**
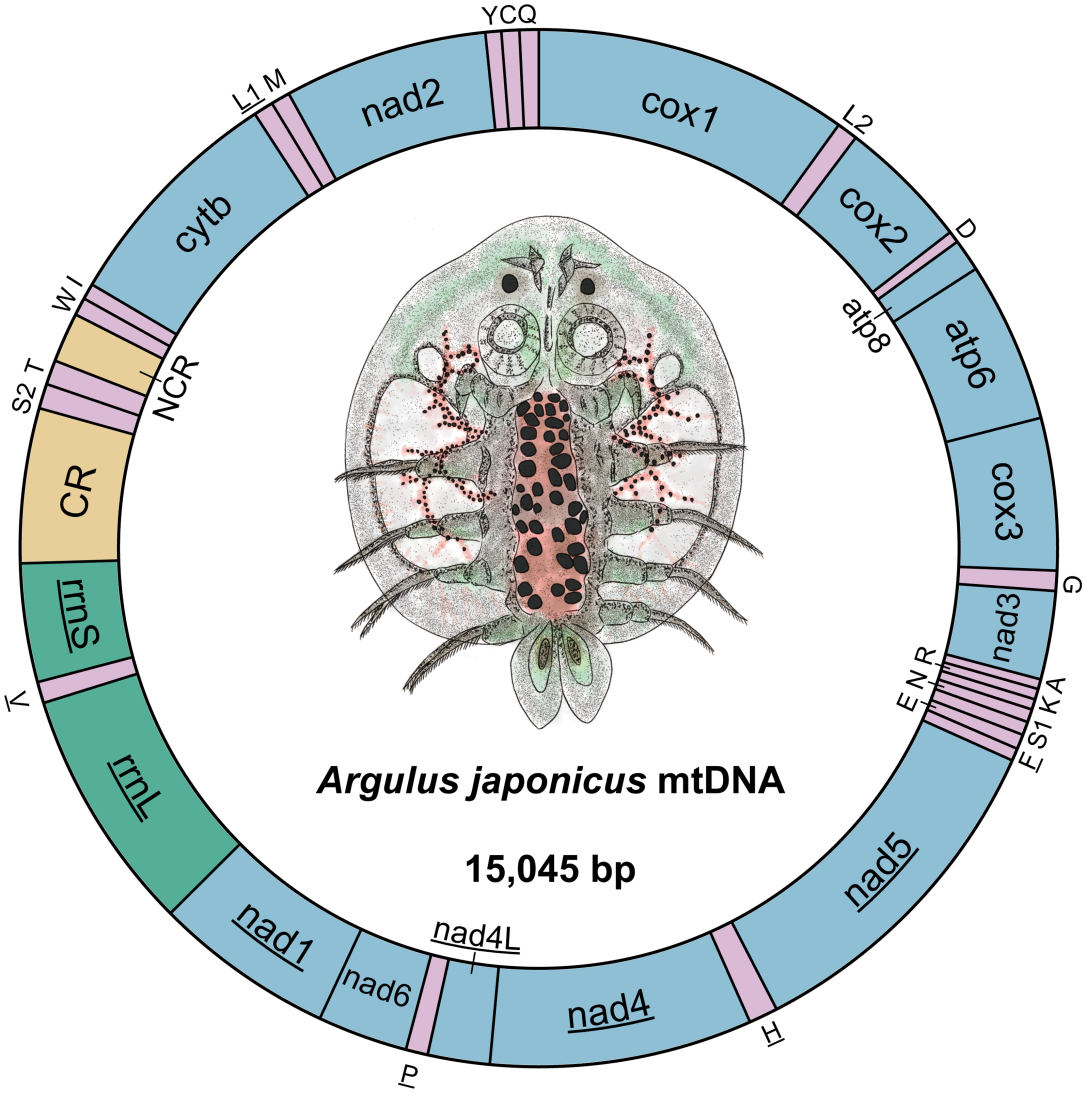
Circular representation of the *A. japonicus* mitogenome. Twenty-two tRNAs are represented by single letters corresponding to their respective amino acid codes. Two leucine genes are distinguished as L1 and L2, and two serine genes are labeled as S1 and S2. The genes located on the N-strand are underlined. NCR denotes the non-coding region, and CR denotes the control region.

The nucleotide sequence of the *A. japonicus* mitogenome was determined to be 15,045 bp in size (Figure 1). It was composed of 37 genes, including 13 PCGs (1 subunit of cytochrome c-ubiquinol oxidoreductase, cytb; 2 subunits of the adenosine triphosphatase synthase, atp6, and atp8; 3 subunits of cytochrome c oxidase, cox1-3; and 7 subunits of nicotinamide dehydrogenase, nad1-6, and nad4L), 22 transfer RNAs (tRNAs) (2 coding for leucine and 2 coding for serine), and 2 ribosomal RNAs (rRNAs) (small [rrnS] and large [rrnL] subunits), consistent with those found in other crustaceans for which complete mitogenomes are available (11, 37, 44–49). Twenty-three genes (14 tRNAs and 9 PCGs) were located on the J-strand, and the remaining 14 genes (8 tRNAs, 4 PCGs, and 2 rRNAs) were observed to be located on the N-strand (Table 1). Further, some genes were found to overlap with each other, and there were a total of 10 overlapping nucleotides observed between genes, as shown in Figure 1. Interestingly, two 7-bp overlaps were found between atp8 and atp6 and between nad4 and nad4L, respectively, similar to those reported in the mitogenome of the intertidal acorn barnacle *Tetraclita serrata* (11). Moreover, eight overlaps ranging from 1 to 2 bp were found to be *Argulus*-specific in comparison to other crustacean mitogenomes (44–49). Two non-coding regions ranging from 316 to 647 bp were also present in the *A. japonicus* mitogenome, with the longest one speculated as the control region (647 bp) and located between rrnS and trnK (Table 1), as described previously in crustacean species, such as *Argulus americanus*, *Armillifer armillatus*, and *Speleonectes tulumensis* (44–49). Consequently, the *A. japonicus* mitogenome was deemed to be compact due to more overlapping spacers (44 bp) and intergenic regions (38 bp) than other crustacean mitogenomes reported thus far (44, 46, 47, 49).

**Table 1:** Organization of the complete *A. japonicus* mitogenome.

**Gene**	**Location and Size (bp)**			**Codon**		**Intergenic nucleotides (bp)**
	**Location**	**Strand**	**Size**	**Start codon**	**Stop codon**	
cox1	1–1,537	J	1,537		T	0
trnL2-UUR	1,538–1,605	J	68			0
cox2	1,606–2,278	J	673	ATG	T	0
trnD	2,279–2,339	J	61			0
atp8	2,340–2,495	J	156	ATC	TAA	–7
atp6	2,489–3,149	J	661	ATG	T	0
cox3	3,150–3,933	J	784	ATG	T	0
trnG	3,934–3,994	J	61			0
nad3	3,995–4,336	J	342	ATC	TAA	−2
trnA	4,335–4,395	J	61			−2
trnR	4,394–4,454	J	61			-2
trnK	4,453–4,522	J	70			0
trnN	4,523–4,581	J	59			0
trnS1-UCU	4,582–4,649	J	67			−1
trnE	4,649–4,711	J	63			−1
trnF	4,710–4,772	N	62			−1
nad5	4,772–6,413	N	1,642	ATA	T	0
trnH	6,414–6,474	N	61			0
nad4	6,475–7,750	N	1,276	ATG	T	−7
nad4L	7,744–8,040	N	297	ATT	TAG	35
trnP	8,076–8,136	N	61			0
nad6	8,137–8,583	J	447	ATT	TAG	16
nad1	8,600–9,508	N	909	ATA	TAG	0
rrnL	9,509–10,559	N	1,051			0
trnV	10,560–10,623	N	64			0
rrnS	10,624–11,269	N	646			-
Control region	11,270–11,916		647			-
trnS2-UGA	11,917–11,976	J	60			50
trnT	12,027–12,086	J	60			-
non-coding region	12,087–12,402		316			-
trnI	12,403–12,463	J	61			−1
trnW	12,463–12,525	J	63			14
cytb	12,540–13,681	J	1,142	ATA	TA	5
trnL1-UAG	13,687–13,749	N	63			58
trnM	13,808–13,871	J	64			15
nad2	13,887–14,852	J	996	ATC	TAA	3
trnY	14,856–14,916	N	61			7
trnC	14,925–14,985	N	61			−2
trnQ	14,984–15,051	N	68			11

### 3.2 Nucleotide composition and codon usage

**Figure 2:**
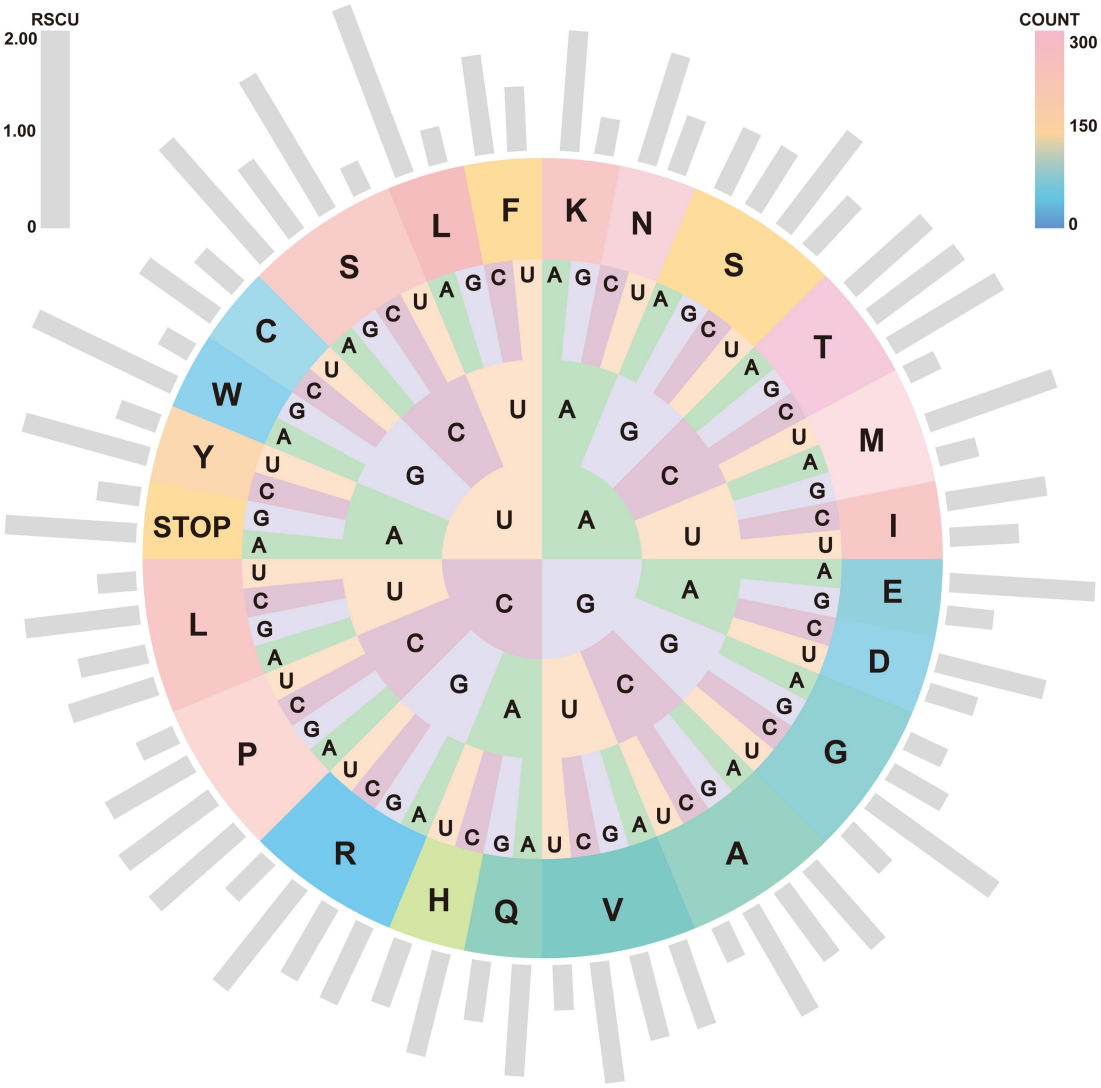
RSCU and codon numbers in the *A. japonicus* mitogenome. Outside gray bars depict the RSCU for individual codons. From inside to outside circles: first, second, and third codon positions in the codon and their coding target amino acids are represented by their abbreviations. Colors from pink to orange and then to blue indicate the different amino acid counts.

The nucleotide composition of the *A. japonicus* mitogenome was 36.93% A, 34.71% T, 18.54% C, and 9.82% G, which led to the conclusion that A predominated while G was the least favored. It was notable that the 71.64% A + T and 28.36% G + C contents of the concatenated PCGs accounted for the largest proportions in this nucleotide composition when compared to those of rRNAs and tRNAs. Furthermore, the *A. japonicus* mitogenome also exhibited significant C-skew (GC skew = −0.307), similar to other Arguloida and Porocephalida species within Crustacea (44, 47, 49). Such nucleotide bias had an appreciable effect on both codon usage patterns and relative synonymous codon usage (RSCU). RSCU and codon counts in the *A. japonicus* mitogenome were computed and shown in Figure 2. It became apparent that the most frequently used codon was UUA (RSCU = 2.07), followed by UCA (RSCU = 1.82) and GUA (RSCU = 1.62). Correspondingly, the most frequently used amino acids included Leu (Count = 449), Lys (Count = 284), Phe (Count = 260), and Asn (Count = 255). Besides, ATG served as the predominant start codon for *A. japonicus* PCGs (cox2, cox3, atp6, and nad4), followed by ATC (atp8, nad3, and nad2), ATA (cytb, nad1, and nad5), and ATT (nad4L and nad6). Among these PCGs, six genes were deduced to utilize TAG (nad1, nad4L, and nad6) or TAA (nad2, nad3, and atp8) as the stop codons, and the remaining seven genes were anticipated to end with an incomplete codon, such as T (atp6, cox1-3, nad4, and nad5) or TA (cytb). A similar usage of the incomplete stop codons was also observed in other crustaceans (44, 47, 49), and they were presumably converted into complete ones by post-transcriptional polyadenylation during mRNA maturation (50, 51).

### 3.3 PCG variability and substitution ratios

In order to understand the evolutionary divergence between *A. japonicus* and other crustacean species, the nucleotide and amino acid sequence differences of 13 PCGs were measured. As shown in Supplementary Table S3, it appeared evident that *A. japonicus* shared the highest sequence identities with the congeneric *A. americanus* (49) and the lowest sequence identities with the cephalocarid *C. hyperboreus* (52). Among these PCGs, the cox1 gene was further determined to have the highest nucleotide (67.12%) and amino acid (70.93%) sequence identities, in contrast, the nad6 gene was determined to have the lowest nucleotide (23.09%) and amino acid (39.20%) sequence identities, to some extent, suggesting that the cox1 might be the slowest evolving and most conserved gene while the nad6 was the least conserved gene among crustacean mitogenomes. Such variability also implied the potential of the cox1 gene as a molecular marker for species- and population-level genetic investigations, in contrast with the nad6 gene as a DNA barcode for species identification and differentiation among crustaceans. Indeed, the cox1 gene has been regarded as a new marker for studying genetic variation among *A. japonicus* individuals from China, Egypt, and Syria (31); by contrast, the nad6 gene has been used as a DNA barcode for the identification of parasitic arthropods, including species of Tabanidae (53), Siphonostomatoida (54), and Arguloida (16).

**Figure 3:**
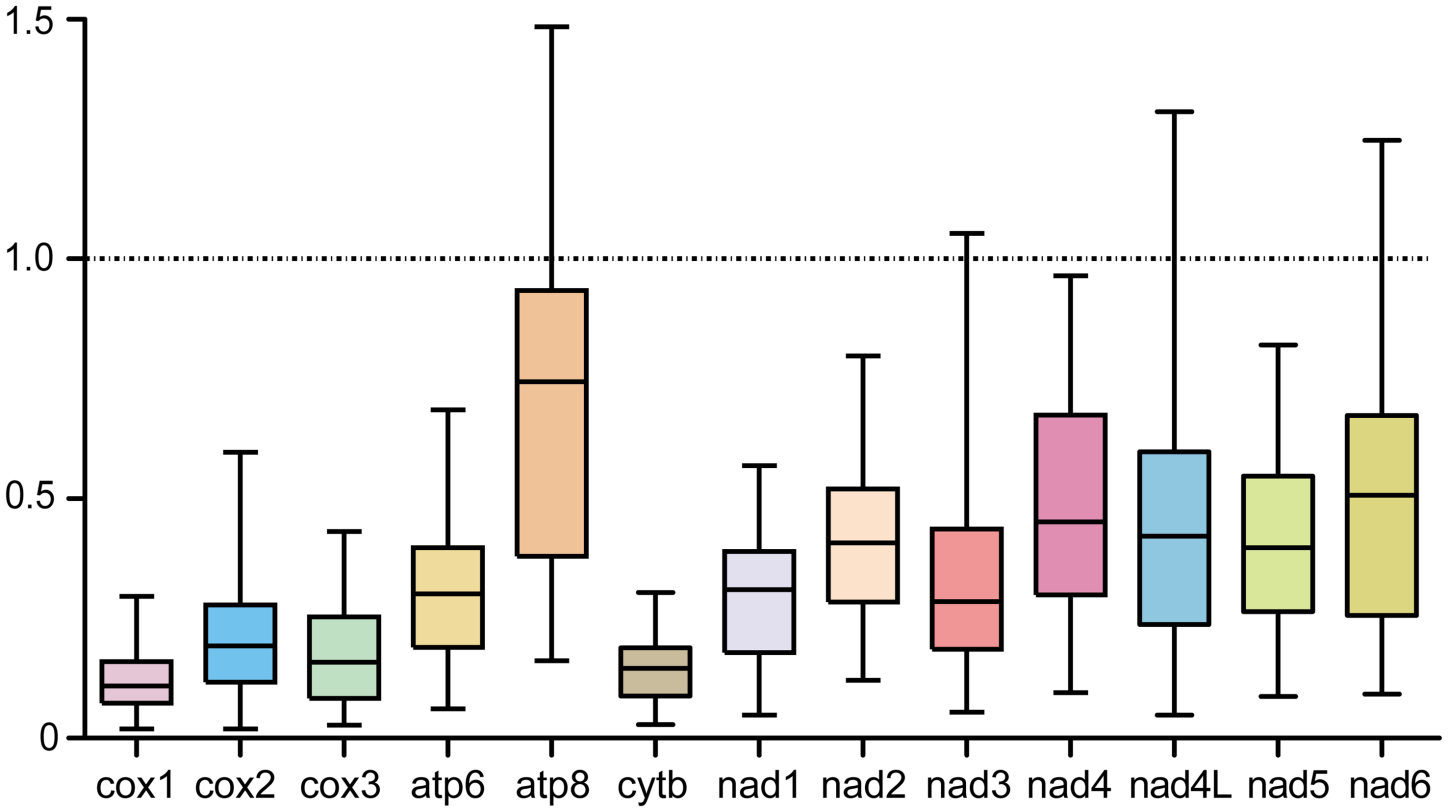
Evolutionary rates of PCGs between *A. japonicus* and other crustaceans. The rates of non-synonymous substitutions (Ka) and synonymous substitutions (Ks) and the ratio of Ka/Ks are calculated for each PCG.

In parallel with the sequence variability, Ka, Ks, and Ka/Ks values were also calculated for PCGs in order to estimate their evolutionary rates, and the ratio of Ka/Ks was simultaneously used for assessing the selective pressure of PCGs and indicating negative or purifying selection when Ka/Ks < 1, neutral mutation when Ka/Ks = 1, and positive or diversifying selection when Ka/Ks > 1 (55, 56). As shown in Figure 3, the atp8 gene among the 13 PCGs showed the maximum ratio of Ka/Ks (0.618), followed by nad4 (0.426), nad6 (0.421), nad4L (0.387), nad2 (0.372), nad5 (0.371), nad3 (0.282), atp6 (0.280), nad1 (0.266), cox2 (0.176), cox3 (0.147), cytb (0.132), and cox1 (0.106). Nevertheless, these ratios were all below 1, supporting a negative or purifying selection that acted on crustacean PCGs during their evolutions. Of course, to further test this certainty, it is still imperative to obtain evidence from the nuclear genomes.

### 3.4 Genetic distances

**Figure 4:**
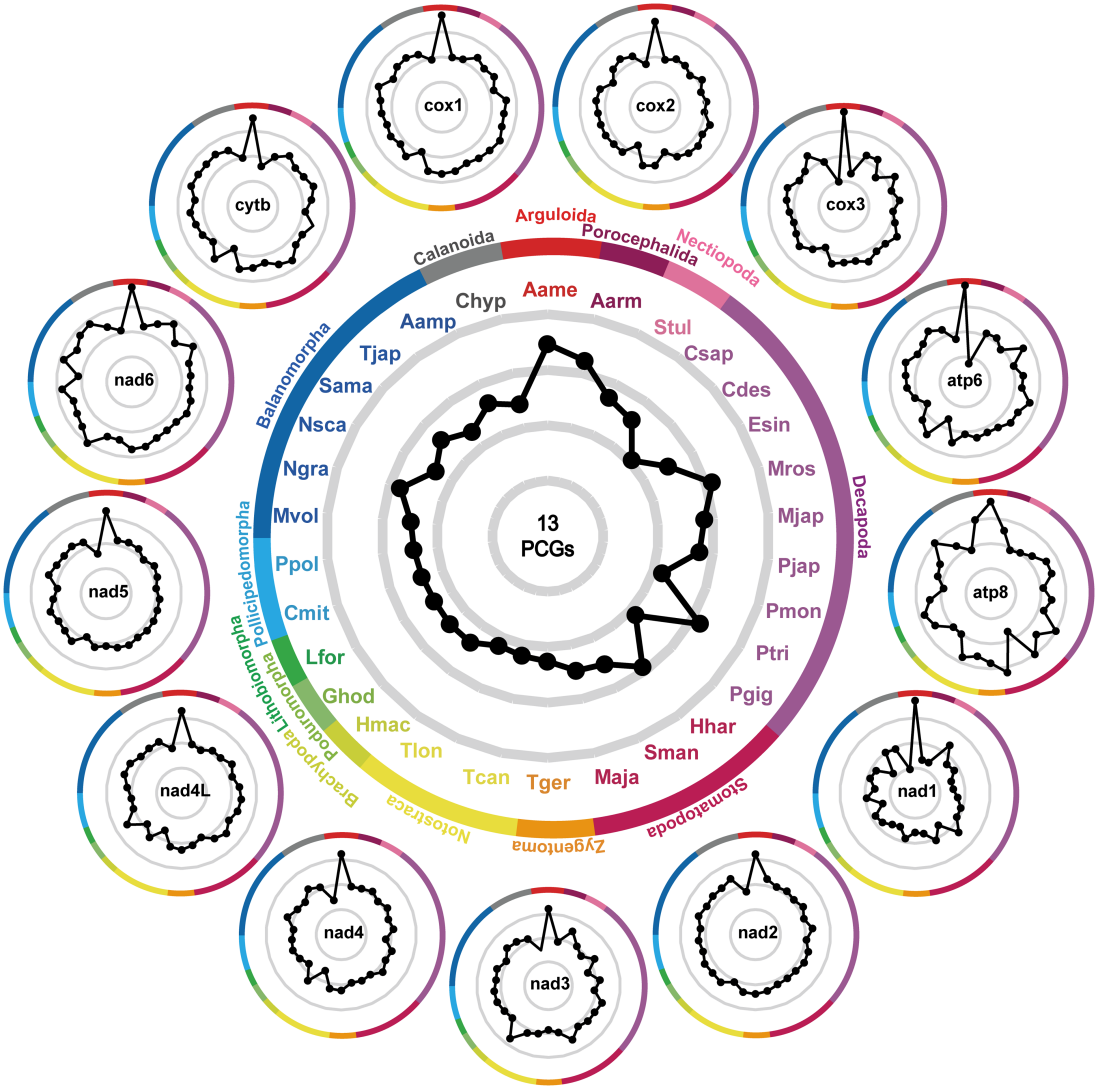
Patterns of K2P distances between *A. japonicus* and other related 30 crustaceans. The gray lines represent identical K2P distances radiating from the center. Black dots signify the relative K2P distances between *A. japonicus* and other crustaceans. Dots positioned closer to the edge of the pattern denote a diminished K2P distance between *A. japonicus* and the respective species. The central figure is generated on the basis of the concatenated amino acid sequences of 13 PCGs, and the outside individual phenogram is generated on the basis of the single PCG. The abbreviations Aamp, Aame, Aarm, Chyp, Csap, Cmit, Cdes, Esin, Ghod, Hmac, Lfor, Mros, Mjap, Mvol, Maja, Hhar, Ngra, Nsca, Pjap, Pmon, Ppol, Ptri, Pgig, Stul, Sman, Sama, Tjap, Tger, Tcan, Tcan, and Tlon represent *Amphibalanus amphitrite*, *Argulus americanus*, *Armillifer armillatus*, *Calanus hyperboreus*, *Callinectes sapidus*, *Cherax destructor*, *Eriocheir sinensis*, *Gomphiocephalus hodgsoni*, *Hutchinsoniella macracantha*, *Lithobius forficatus*, *Macrobrachium rosenbergii*, *Marsupenaeus japonicus*, *Megabalanus volcano*, *Harpiosquilla harpax*, *Nobia grandis*, *Notochthamalus scabrosus*, *Panulirus japonicus*, *Penaeus monodon*, *Pollicipes polymerus*, *Portunus trituberculatus*, *Pseudocarcinus gigas*, *Speleonectes tulumensis*, *Squilla mantis*, *Striatobalanus amaryllis*, *Tetraclita japonica*, *Tricholepidion gertschi*, *Triops cancriformis*, and *Triops longicaudatus*, respectively.

In addition to evolutionary divergence, we calculated the interspecific genetic distances between *A. japonicus* and other crustaceans using single or concatenated PCGs (Figure 4). It was clear that regardless of single or concatenated PCG datasets, the minimum K2P-based genetic distances were consistently present between *A. japonicus* and *A. americanus* (0.227–0.438 for single PCG datasets and 0.594 for concatenated PCG datasets). In contrast, the maximum genetic distances present for *A. japonicus* and *C. hyperboreus* are 0.366–0.981 for single PCG datasets and 0.641 for concatenated PCG datasets, once again confirming that *A. japonicus* was closely related to *A. americanus* but diverged from *C. hyperboreus* (57). Comparisons of genetic distance structures showed that the values of the cox1-based K2P genetic distances were all significantly smaller than those of other single PCG and concatenated PCGs, in agreement with the aforementioned result, in which the cox1 was regarded as the most conserved gene among crustacean species (29). Perhaps this conclusion can be further validated when additional crustacean mitogenomes become available, especially from the genus *Argulus*.

### 3.5 Phylogeny

**Figure 5:**
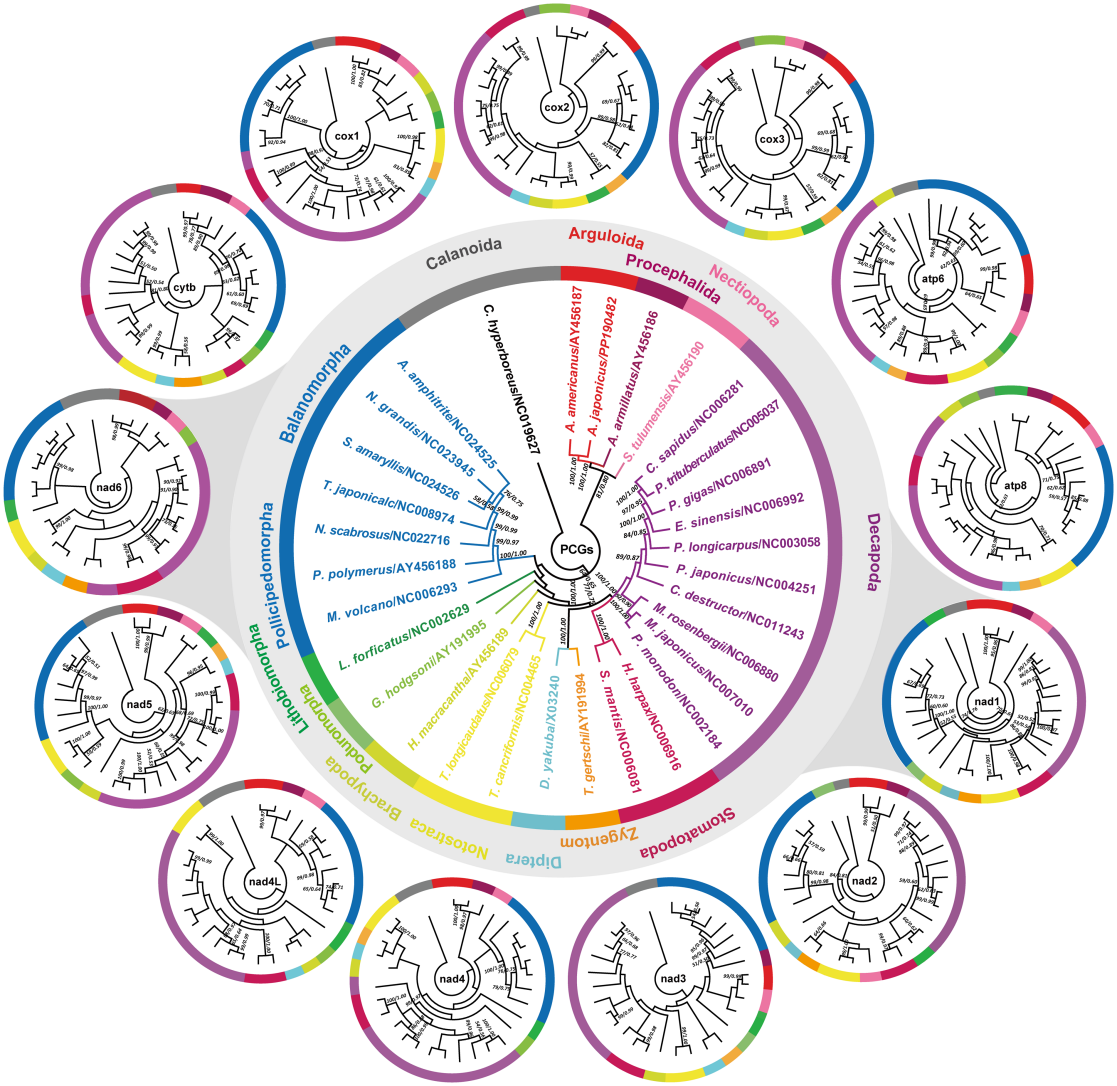
Phylogenetic relationships among crustacean species. Phylogenies are inferred on the basis of the amino acid sequences of single or concatenated PCGs of *A. japonicus* and other crustacean species using MP and BI methods. One Calanoida species (*C. hyperboreus*) is used as the outgroup and included in each tree. The central tree is reconstructed based on the concatenated amino acid sequences of 13 PCGs, and the outside 13 individual trees are reconstructed based on the single PCG. The single PCG-based trees that share the same topology as the concatenated PCG-based trees are highlighted with gray shading. The numbers along the branches indicate bootstrap values/posterior probabilities resulting from different analyses in the order MP/BI. Values less than 50/0.5 are not displayed.

The available *A. japonicus* mitogenome provided us with an opportunity to study the phylogenetic relationships of *A. japonicus* in the family Argulidae and of the family Argulidae within Crustacea. Congruent with previous phylogenetic analyses (11, 44, 47, 49), phylogenies in this study were inferred from the concatenated amino acid sequence dataset derived from 13 PCGs. As shown in Figure 5, the identical phylogenetic tree (MP/BI) robustly supported the distinct classification of Porocephalida, Nectiopoda, Decapoda, Stomatopoda, Diptera, Zygentoma, Notostraca, Brachypoda, Poduromorpha, Lithobiomorpha, Balanomorpha, and Pollicipedomorpha within Crustacea, each as a monophyletic group. It was also noteworthy that there was a closer relationship between species of the families Argulidae (Arguloida) and Armilliferidae (Porocephalida) compared to other crustacean species, and further, *A. japonicus* and *A. americanus* were determined to be more closely related to each other than to others within the family Argulidae, consistent with recent nuclear and mt DNA-based phylogenetic conclusions (57, 58). Additionally, species from Nectiopoda, Decapoda, Stomatopoda, Diptera, Zygentoma, Notostraca, Brachypoda, and Poduromorpha were individually clustered together as paraphyletic relationships in Crustacea, in accordance with the results of morphological and molecular biology studies (11, 59–63). Nevertheless, species from Lithobiomorpha exhibited a poor affinity for species from Balanomorpha and Pollicipedomorpha. Perhaps, a larger study of the evolutionary relationships among taxa within Crustacea is still needed by sequencing additional crustacean parasites, especially those from the order Lithobiomorpha.

Furthermore, single PCG-based phylogenies were also achieved to identify the potential genetic marker candidates for molecular diagnostics and phylogenetic studies in Crustacea. As shown in Figure 5, it was apparent that although most PCGs exhibited diverse topologies, the classification relationships between Arguloida and Porocephalida as well as between Diptera and Zygentoma were stable in the cytb-, cox1-, nad1-, nad2-, nad4-6-, and atp6-based analyses, consistent with findings reported in previous studies (11, 44, 47, 49, 64). Further comparisons of these tree structures revealed that the nad1 and nad6 genes shared a similar phylogenetic topology with that of the genome-based phylogeny in comparison to other PCGs, suggesting their potential as the most suitable genetic markers instead of the complete mitogenomes for molecular diagnostic, systematic, and evolutionary biological studies of *A. japonicus* and related crustacean species. Of course, their marker effectiveness remains to be further tested by using more crustacean mitogenomes. Therefore, there is no doubt that the concatenated PCG datasets might be the optimal marker choice for evolutionary and phylogenetic studies of crustacean species.

## 4 Conclusion

In the present study, we presented a comprehensive characterization of the evolutionary blueprint of *A. japonicus* by sequencing the complete mitogenome and its genetic comparisons with other related species. Comparative genomics indicated that among PCGs, cox1 was the most conserved gene, whereas nad6 was the most varied gene. Genome- and single-gene-based phylogenies supported a close relationship between *A. japonicus* and *A. americanus* within the family Argulidae. Further phylogenetic relationship comparisons suggested potential applications of the nad1 and nad6 genes as novel genetic markers for evolutionary and phylogenetic studies of crustacean species. These results are expected to have implications for molecular diagnostic methods, epidemiological investigations, and the control of *A. japonicus*. The information will also be important in the refinement of the phylogenetic relationships within Crustacea and in accumulating valid markers for systematic, population genetic, and evolutionary biological studies of parasitic crustaceans of socio-economic importance.

## Data availability statement

The data presented in the study is deposited in the GenBank repository, accession number PP190482.

## Ethics statement

The manuscript presents research on animals that do not require ethical approval for their study.

## Author contributions

LW: Conceptualization, Data curation, Formal analysis, Investigation, Methodology, Software, Validation, Visualization, Writing – original draft. ZH: Conceptualization, Data curation, Formal analysis, Investigation, Methodology, Software, Validation, Visualization, Writing – original draft. ZW: Conceptualization, Data curation, Formal analysis, Investigation, Methodology, Software, Validation, Visualization, Writing – original draft. PZ: Formal analysis, Methodology, Software, Visualization, Writing – original draft. GW: Formal analysis, Software, Visualization, Writing – original draft. XF: Data curation, Visualization, Writing – original draft. JH: Formal analysis, Visualization, Writing – original draft. RW: Formal analysis, Writing – original draft. HW: Conceptualization, Project administration, Supervision, Writing – original draft, Writing – review & editing. YX: Conceptualization, Funding acquisition, Investigation, Project administration, Supervision, Writing – original draft, Writing – review & editing.

## Footnotes

^1^ https://macvector.com/

^2^ https://www.dnastar.com/

^3^ https://tcoffee.crg.eu/

^4^ https://www.biologiaevolutiva.org/jcastresana/Gblocks.html

^5^ https://nbisweden.github.io/MrBayes/download.html

^6^ https://directory.fsf.org/wiki/TreeView_X
